# Fano factor constancy and scale-invariant sampling in recurrent networks with probabilistic synapses

**DOI:** 10.1186/1471-2202-13-S1-P84

**Published:** 2012-07-16

**Authors:** Ruben Moreno-Bote

**Affiliations:** 1 Foundation Sant Joan de Deu, Parc Sanitari Sant Joan de Deu, 08950 Esplugues de Llobregat, Barcelona, Spain

## 

Spiking activity in cortex is highly variable both spontaneously and during stimulation. It has been observed that this neuronal variability has the remarkable property that the Fano factor of the neuron´s spike counts (variance/mean) is constant over broad ranges of their firing rates, a property that we call “Fano factor constancy”. Realistic neuronal networks models in the balanced regime do not lead to Fano factor constancy over broad ranges of firing rate unless their parameters are fine-tuned, posing a problem.

What mechanism could lead to Fano factor constancy? One well-documented source of variability in cortex is synaptic noise. However, whether synaptic noise can lead to the observed properties of neuronal variability is currently unknown. To address this problem, we studied analytically a network of excitatory and inhibitory spiking integrate-and-fire neurons connected with arbitrary connectivity matrices. The network was endowed with stochastic synaptic release with fixed probability. The system was solved in the stationary regime when the network is strongly driven by stimuli. To study robustness of the results, we studied numerically networks with short-term synaptic plasticity with stochastic release and with uncompleted saturation, in line with recent *in vivo* experimental results. Here we show that realistic neuronal networks of spiking neurons endowed with probabilistic synaptic release naturally generate constant Fano factors over several orders of magnitude in the neurons´ firing rate. The mechanism does not require fine tuning of the network parameters, but rather it arises naturally from the amplification of the synaptic noise and the neuronal spiking threshold. Other mechanisms that introduce noise in the system like channel noise or constant external noise do not lead to Fano factor constancy.

Probabilistic synaptic release naturally leads to information loss. Then, what could be the role of this form of variability? We show that synaptic noise allows sampling the states of the network and, furthermore, that the Fano factor constancy property implies that the state-sampling dynamics obeys a scale-invariant rule: if the input drive to the network is scaled by some factor, the probability of visiting the states of the network remains constant. This means that, for instance, if the contrast of an ambiguous input image is doubled, the probability of interpreting it in a particular way does not change. This result suggests that a form of Weber-like scale-invariance perception is naturally implemented in neuronal networks with probabilistic synapses.

Therefore, synaptic noise might not only be the responsible for the type of variability found in cortex, but it also might endow cortical neurons with crucial computational properties to solve inference problems with ambiguous information.

